# Culture-Independent Evaluation of the Appendix and Rectum Microbiomes in Children with and without Appendicitis

**DOI:** 10.1371/journal.pone.0095414

**Published:** 2014-04-23

**Authors:** Hope T. Jackson, Emmanuel F. Mongodin, Katherine P. Davenport, Claire M. Fraser, Anthony D. Sandler, Steven L. Zeichner

**Affiliations:** 1 Sheikh Zayed Institute for Pediatric Surgical Innovation Children's National Medical Center, Washington DC, United States of America; 2 Institute for Genome Sciences, University of Maryland School of Medicine, Baltimore, Maryland, United States of America; 3 Department of Pediatric Surgery, and the Sheikh Zayed Institute for Pediatric Surgical Innovation Children's National Medical Center, Washington DC, United States of America; 4 Center for Cancer and Immunology Research, Children's Research Institute, Children's National Medical Center, Washington DC, United States of America; 5 Departments of Pediatrics and Microbiology, Immunology, and Tropical Medicine George Washington University, Washington DC, United States of America; Universidad Andres Bello, Chile

## Abstract

**Purpose:**

The function of the appendix is largely unknown, but its microbiota likely contributes to function. Alterations in microbiota may contribute to appendicitis, but conventional culture studies have not yielded conclusive information. We conducted a pilot, culture-independent 16S rRNA-based microbiota study of paired appendix and rectal samples.

**Methods:**

We collected appendix and rectal swabs from 21 children undergoing appendectomy, six with normal appendices and fifteen with appendicitis (nine perforated). After DNA extraction, we amplified and sequenced 16S rRNA genes and analyzed sequences using CLoVR. We identified organisms differing in relative abundance using ANOVA (p<0.05) by location (appendix vs. rectum), disease (appendicitis vs. normal), and disease severity (perforated vs. non-perforated).

**Results:**

We identified 290 taxa in the study's samples. Three taxa were significantly increased in normal appendices vs. normal rectal samples: *Fusibacter* (p = 0.009), *Selenomonas* (p = 0.026), and *Peptostreptococcus* (p = 0.049). Five taxa were increased in abundance in normal vs. diseased appendices: *Paenibacillaceae* (p = 0.005), *Acidobacteriaceae GP4* (p = 0.019), *Pseudonocardinae* (p = 0.019), *Bergeyella* (p = 0.019) and *Rhizobium* (p = 0.045). Twelve taxa were increased in the appendices of appendicitis patients vs. normal appendix: *Peptostreptococcus* (p = 0.0003), *Bilophila* (p = 0.0004), *Bulleidia* (p = 0.012), *Fusobacterium* (p = 0.018), *Parvimonas* (p = 0.003), *Mogibacterium* (p = 0.012), *Aminobacterium* (p = 0.019), *Proteus* (p = 0.028), *Actinomycineae* (p = 0.028), *Anaerovorax* (p = 0.041), *Anaerofilum* (p = 0.045), *Porphyromonas* (p = 0.010). Five taxa were increased in appendices in patients with perforated vs. nonperforated appendicitis: *Bulleidia* (p = 0.004), *Fusibacter* (p = 0.005), *Prevotella* (p = 0.021), *Porphyromonas* (p = 0.030), *Dialister* (p = 0.035). Three taxa were increased in rectum samples of patients with appendicitis compared to the normal patients: *Bulleidia* (p = 0.034), *Dialister* (p = 0.003), and *Porphyromonas* (p = 0.026).

**Conclusion:**

Specific taxa are more abundant in normal appendices compared to the rectum, suggesting that a distinctive appendix microbiota exists. Taxa with altered abundance in diseased and severely diseased (perforated) samples may contribute to appendicitis pathogenesis, and may provide microbial signatures in the rectum useful for guiding both treatment and diagnosis of appendicitis.

## Introduction

The physiologic function of the human appendix is largely unknown. Some hypotheses hold that the appendix plays a critical role in the education, development and maturation of the immune system [1], [2]. Other studies suggest that the appendix may serve as a reservoir for beneficial components of the GI microbiome that can repopulate the GI tract following acute illness [3], [4]. While these theories are intriguing, the detailed function of the appendix remains poorly understood, but likely involves interactions between the lymphoid tissue that exists in abundance in the appendix and the microbiota contained within the appendix. Shifts in the appendiceal microbiota are believed to play a key role in the pathophysiology of acute appendicitis, a common pediatric and adult disorder.

Acute appendicitis is a classic disease of the modern medical era. It is a paradigm for the application of increasingly sophisticated diagnostic, medical, and surgical technology. Appendicitis was one of the first acute non-traumatic disorders effectively cured by surgery [5]. Later, once it became available, antimicrobial therapy demonstrated great efficacy for the complications of perforated appendicitis [6]. Appendicitis affects ∼77,000 patients/year in the US, with annual costs estimated at $680 million/year [7]. The lifetime risk of appendicitis has been estimated at 7% [8], with peak incidence occurring between 10 and 30 years of age, although as patients age, the characteristics of the disease may be clouded by other similarly presenting disorders. The incidence of appendicitis varies in different populations, in different regions, and over time [8], [9], [10]. The changing incidence has been attributed to a variety of environmental and behavioral factors that include general hygiene, parasitic infections, enteric infections resulting in GI lymphoid hyperplasia and variations in consumption of dietary fiber, but the definitive causes of appendicitis remain poorly understood.

The pathogenesis of appendicitis is classically thought to result, in part, from obstruction of the appendiceal lumen. Obstruction has been attributed to lymphoid hyperplasia, anatomic position, tumors and fecaliths, which are found in 11–52% of patients with acute appendicitis [11], [12], [13]. Obstruction is then thought to lead to an accumulation of undrained secretions, alteration and overgrowth of appendiceal microbes, compromised perfusion, and epithelial damage [9], [14], [15], [16]. The precise sequence of events is not definitively established, but most authors count microbial overgrowth or distortion of the appendiceal microbial flora as key elements of the pathogenic cascade.

Several reports over many years have described the bacteriology of appendicitis [17], [18], [19], [20], [21], [22], [23]. These studies initially focused on using conventional culture techniques to study the bacteriology of the diseased appendix, the peritoneum and the surgical wound following appendiceal rupture. While some information has been published concerning the bacteriology of the diseased appendix, the bacteriology of the normal appendix is even less well understood, particularly at the complete microbiome level using culture-independent approaches. One study of quantitative bacterial colony counts using conventional techniques showed no differences between normal and inflamed appendices [20]. Another study identified *Bilophilia sp* as a new microbe from cultures of appendicitis samples, while another study identified *Fusobacterium sp* as the major microbe responsible for acute appendicitis using rRNA-based fluorescence in situ hybridization (FISH) [21], [23]. A recent study [22] provided a culture-independent survey of the appendix using gene sequencing. This study examined seven patients presenting with signs and symptoms of appendicitis. The study found that the notable taxa in the appendix were *Firmicutes, Proteobacteria, Bacteroidetes, Actinobacteria, and Fusobacteria*, but only looked at those patients with suspected appendicitis. None of these studies have been extensively replicated.

While traditional, culture-based techniques have been used to characterize microbial populations [24], [25], these conventional culture studies have not yielded conclusive information about the contribution of the appendix microbiota to health and disease. This is likely, at least in part, because standard culturing approaches do not furnish a complete picture of prokaryotic diversity, as more than 90–99% of the microbes are not culturable by standard techniques [26]. Based on the observation that 16S ribosomal RNA (rRNA) genes are highly conserved within species and among species of the same genus, knowledge of microbial diversity has expanded enormously as a result of applying recently developed 16S rRNA-gene sequencing-based culture-independent approaches [27], [28], [29].

We conducted a pilot study using a culture-independent 16S rRNA gene-based examination of the appendix microbiota in pediatric patients, with corresponding matched rectal samples from each patient. We studied both patients diagnosed as having appendicitis and patients undergoing appendectomy incidental to abdominal surgery for another indication. We catalogued and compared the relative abundance of bacterial genera in both healthy and diseased appendix samples, and in matched rectal samples. We found that the appendix microbiota significantly differs from the rectal microbiota, that the microbiota of the diseased appendix differs from the microbiota of the healthy appendix, and further that the microbiota of the appendix in appendicitis patients with a perforated appendix differs from the microbiota of the appendix in non-perforated appendicitis. Interestingly, we also found that the bacterial profile observed in the rectum differed between patients with normal appendices and appendicitis patients. Therefore, it may be possible that culture-independent microbial identification, particularly in rectal samples, could improve management of appendicitis, and suggest a new approach for possible appendicitis molecular diagnostics.

## Materials and Methods

### Study Participants

**Figure 1:**
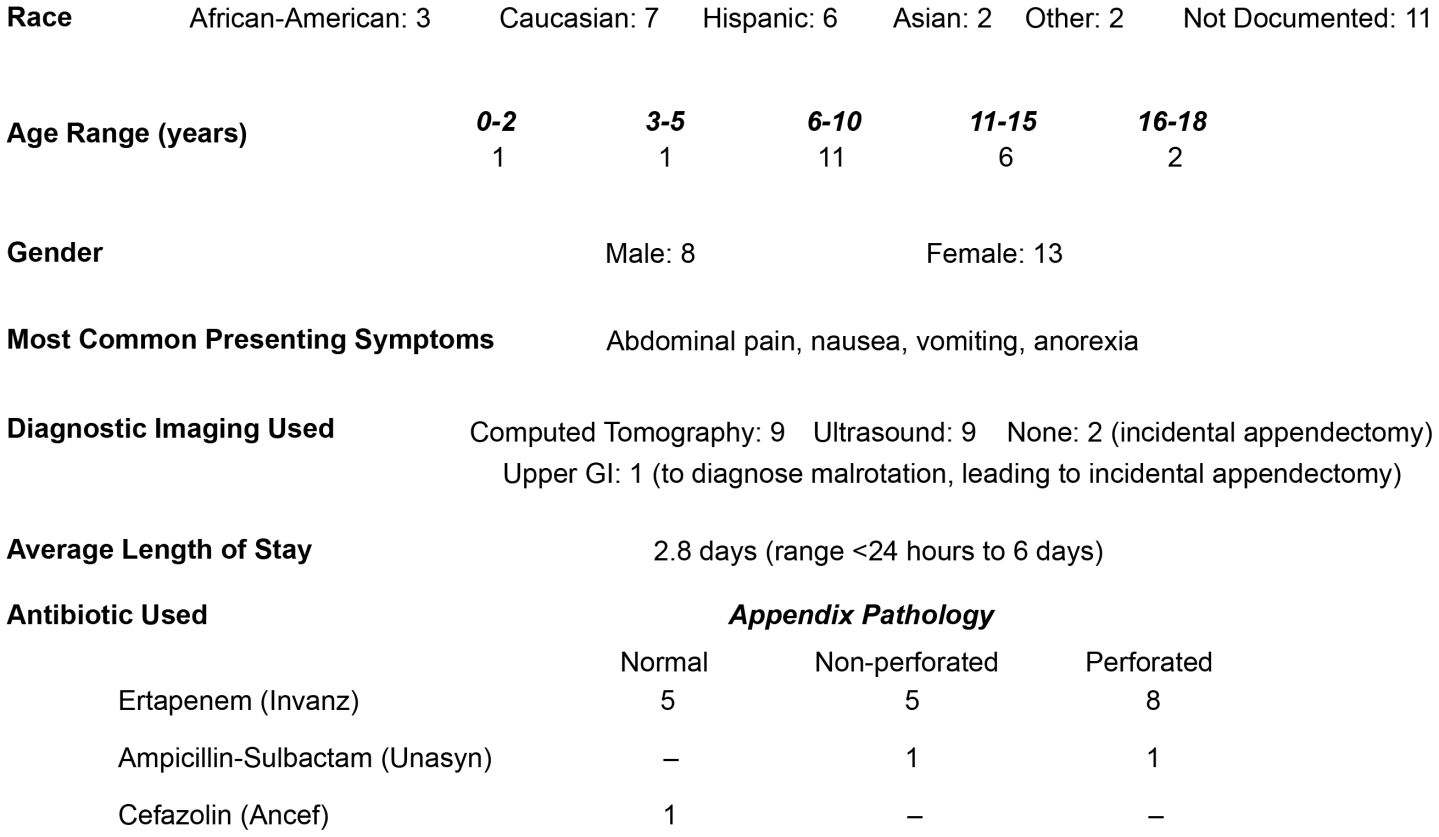
Participant demographics and clinical data. Of note, in those patients where an upper GI or no diagnostic imaging was performed, appendicitis was not the preoperative diagnosis and the appendectomy was incidental.

Following approval of the Children's National Medical Center IRB, we collected, with written consent from the parent or guardian, appendix and rectal swabs from 21 children undergoing appendectomy. The ages of participants ranged from 5 months to 18 years (8 males and 13 females) (Figure 1). Six patients had normal appendices and fifteen patients had appendicitis, nine of which were categorized as perforated based on pathology report. Seventeen patients underwent an appendectomy with an established or presumed diagnosis of acute appendicitis. The remaining four patients received an appendectomy incidental to another condition. Incidental appendectomies are common practice in the pediatric population, and in some situations considered a standard-of-care to help clarify a diagnosis or eliminate appendicitis from the differential diagnosis in a patient with a history of abdominal complaints or abnormal gastrointestinal anatomy. The most common presenting symptoms for the group of patients with appendicitis were nausea, vomiting, anorexia and abdominal pain. All participants, including patients who underwent incidental appendectomies, were given antibiotics within 24 hours of arrival at the hospital up until the time of surgery. See Figure 1 for a list of patient demographics and clinical data, including the antibiotics given to the patients.

### Sample Acquisition

Once each patient was taken to the operating room and placed under anesthesia, an internal swab of the patient's rectum was performed and stored using the Copan ESwab (Copan Diagnostics) collection and preservation system. After the appendix was resected, it was inspected and opened with Metzenbaum scissors. The full length of the opened appendix was swabbed with an ESwab and the swab was then placed in the transport container. The specimens were stored at −80 C until analysis.

### Pathological Diagnosis

The diagnosis of appendicitis was established with pathological reports, which included both macro and microscopic examination of the appendix. Operative reports were reviewed for surgeon commentary on the macroscopic condition of the appendix (inflamed, injected, grossly perforated, normal). The clinical assessment of appendicitis of the surgeon and the pathologic diagnosis of appendicitis agreed in all cases.

### DNA Extraction and 16S rRNA Gene Sequencing

Total genomic DNA was extracted using a protocol developed at the University of Maryland Institute for Genome Sciences and previously described [30]. Briefly, samples were thawed on ice, incubated in an enzymatic cocktail containing lysozyme, mutanolysin, proteinase K and lysostaphin, after which the microbial cells were lysed using bead beating with the FastPrep instrument (MBio, Santa Ana, CA). The DNA was then further extracted and purified using the Zymo Fecal DNA kit (Zymogen).

The variable regions V1–V3 of the 16S rRNA gene were PCR amplified using barcoded 27F and 338R 16S primers, as described previously [30]. Negative controls without a template were included for each barcoded primer pair. The presence of PCR amplicons was then confirmed by gel electrophoresis on a 2% agarose gel and staining with ethidium bromide. PCR products were quantified using the Quant-iT PicoGreen dsDNA assay, and equimolar amounts (100 ng) of PCR amplicons were pooled prior to pyrosequencing [30]. This 16S amplicon pool was sequenced by 454 FLX Titanium sequencing technology using 454 Life Sciences primer A by the Genomics Resource Center at the Institute for Genome Sciences, University of Maryland School of Medicine, using protocols recommended by the manufacturer as amended by the Center.

### 16S sequence analysis and Statistical Analyses

The pipeline CloVR-16S version 1.1 within the CLoVR system (www.CLoVR.org) [31] was used to bin the raw 16S reads using the sample-specific barcode sequences, then trim the barcode and primer sequences, and process the resulting sequences for phylogenetic analyses (for more information about the CLoVR-16S workflow, see http://clovr.org/methods/clovr-16s/). The average read length after barcode and primer trimming was 368 bp. Statistical analysis of differentially abundant bacterial taxa in the 16S rRNA sequence dataset was performed using the METASTATS tool within CLoVR (http://clovr.org/docs/metastats/). We identified organisms that differed significantly (ANOVA, p<0.05) in relative abundance by anatomic location (appendix vs. rectum), disease state (appendicitis vs. normal) and disease severity (perforated vs. non-perforated).

The 16S sequencing data for all the samples analyzed in this study was submitted to the Sequence Read Archive (SRA; http://www.ncbi.nlm.nih.gov/Traces/sra/), under accession SRP035179.

## Results

In this pilot study aimed at characterizing the microbiota associated with the appendix, matched appendix and rectal swabs were collected from 21 children (age range: 5 months–18 years), six with normal appendices and 15 with appendicitis, out of which nine were perforated. A total of 42 samples were processed and DNA extracted. rRNA gene sequences were PCR amplified using the 27F and 338R bacterial primers, and the PCR amplicons were sequenced using 454 Titanium pyrosequencing. Out of these 42 samples, 5 samples did not yield enough PCR amplicons, and were subsequently removed from the analysis. From the remaining 37 samples that were successfully PCR-amplified and sequenced, we obtained a total of 325,342 non-chimeric sequences (8,793±3888 reads on average per sample) that were assigned to a total of 13,751 unique operational taxonomic units (OTUs) using a cutoff of 95% sequence identity. The CloVR-16S v. 1.1 (http://clovr.org/methods/clovr-16s/) automated analysis pipeline was used to process the raw 454 pyrosequencing reads, perform taxonomic assignments and calculate microbial diversity and richness indices.

Median values of bacterial community richness and diversity calculated for samples groups are displayed in Table 1 (for individual values by samples, see Figure S1 in File S1). The observed number of OTUs (sobs calculator in mothur) was the highest for the rectal samples, compared to appendix (median values: 446 *vs.* 237 OTUs). Among the rectal samples, the bacterial communities associated with perforated appendicitis were characterized by the highest number of observed OTUs (478 OTUs), compared to non-perforated appendicitis rectal samples (411 OTUs), and rectal samples from subjects with a normal appendix (220 OTUs). Among the appendix samples, the number of observed OTUs was the highest for the appendix samples from subjects with perforated appendicitis (344 OTUs), appendix samples from patients with non-perforated appendicitis had 228 OTUs. Appendix samples from patients with a normal appendix had 237 OTUs. According to these observations, severity in diagnosis of appendicitis seems to be characterized by a greater observed bacterial richness (sobs calculator), for both appendix and rectal bacterial communities.

**Table 1:** Diversity and Richness Estimators (median values).

	*N*	Observed Number of OTUs (sobs calculator)	Chao1 (Richness)	ACE (Richness)	Shannon (Diversity)	Simpson
**Appendix**	20	237	507.621	911.130	2.742	0.150
**Rectal**	17	446	957.621	1371.363	3.534	0.083
**Appendix: Normal**	5	237	463.622	804.885	2.742	0.150
**Appendix: Non Perforated Appendicitis**	6	228	542.096	921.922	2.557	0.178
**Appendix: Perforated Appendicitis**	9	344	750.978	1241.873	3.041	0.100
**Rectal: Normal Appendix**	5	220	373.867	604.679	2.312	0.206
**Rectal: Non Perforated Appendicitis**	6	411	884.560	1363.194	3.779	0.056
**Rectal: Perforated Appendicitis**	6	478	1101.720	1680.009	3.756	0.078

Microbial diversity and richness indices. Median values of bacterial community richness and diversity calculated for samples groups are displayed. The observed number of OTUs was highest for the rectal samples, compared to appendix. Among the rectal samples, samples from subjects with perforated appendicitis had the highest number of observed OTUs (478 OTUs), compared to non-perforated appendicitis (411 OTUs) and subjects with normal appendix (220 OTUs). Similarly, with respect to the appendix samples, samples from subjects with perforated appendicitis had the highest number of observed OTUS (344 OTUs), compared to non-perforated appendicitis and normal appendix (228 and 237 OTUs, respectively). This suggests that severity in diagnosis of appendicitis seems to be characterized by a higher number of observed OTUs, compared to normal appendices. The rectal samples from patients with appendicitis also had a larger number of OTUs than samples from patients with normal appendices. Rectal samples from patients with perforated appendicitis had a larger number of OTUs than samples from patients with non-perforating appendicitis. The Chao1 and ACE richness estimators also showed that sample richness was higher for rectal samples compared to appendix samples. Chao1 and ACE richness estimators also showed greater richness for rectal samples from patients with appendicitis than samples from patients with normal appendices. The richness estimators for the rectal samples were higher for patients with perforating appendicitis than for patients with non-perforating appendicitis. The Shannon diversity index displayed a similar trend with rectal samples displaying a higher diversity compared to appendix samples, and with diversity was highest for samples from patients with the most severe form of appendicitis, for both rectal and appendix samples.

In order to further characterize the bacterial biodiversity of the samples analyzed in this study, richness and diversity estimators were calculated. These ecological indices have classically been used to gain insight about bacterial community structures when using 16S rRNA gene datasets. Community richness, through the use of the Chao1 and ACE estimators, provides information about the estimated number of OTUs present in the samples. Measure of the community diversity, through the use of the Shannon and Simpson indices, provide information about the composition of a given bacterial community, e.g. not only a measure of richness but also take into account the relative abundance of OTUs (or evenness). The Chao1 and ACE richness estimators confirmed the observations previously made using the sobs calculator: sample richness was higher for rectal samples compared to appendix, and for perforated appendicitis rectal and appendix samples compared to normal rectal and appendix samples (Table 1). The Shannon diversity index displayed a similar trend with rectal samples displaying a higher diversity compared to appendix samples, and with diversity being the highest for increasing severity of appendicitis diagnosis, for both rectal and appendix samples (Table 1). The Simpson index showed an opposite trend, as expected.

**Figure 2:**
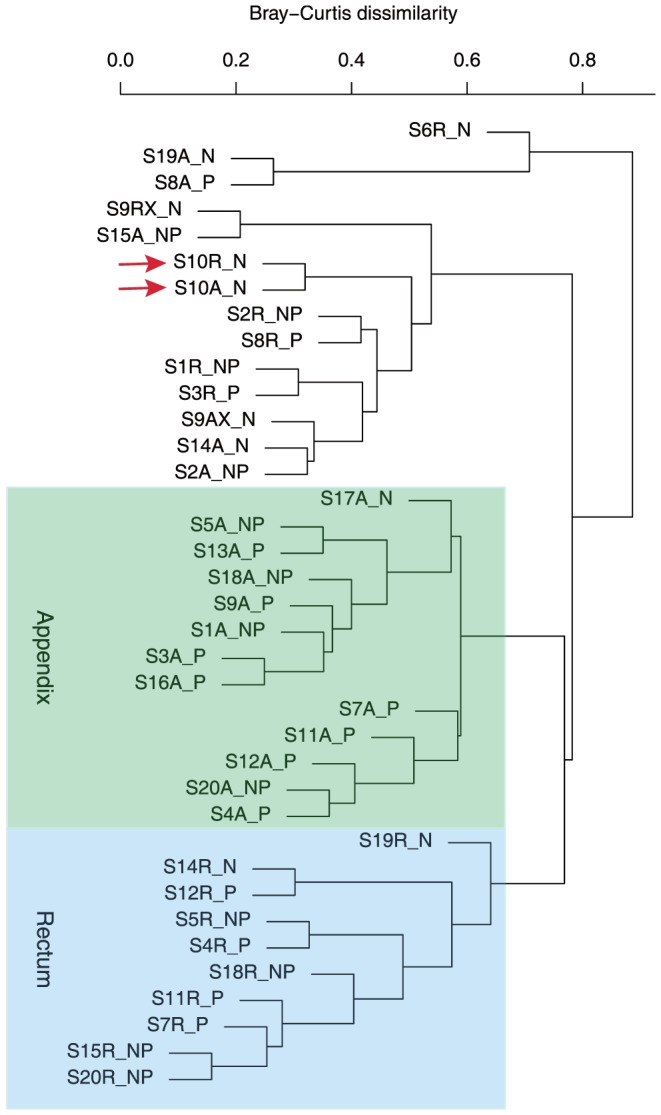
Bray-Curtis Cluster Dendogram. This abundance-weighted measures how similar two communities are in terms of their genus composition using the Bray-Curtis metric [32], [33]. 37 samples were analyzed (16 rectal samples and 20 appendix samples). 13 appendix samples clustered together (green box). A group of 10 rectal samples (blue box) clustered separately from the appendix samples, suggesting that the microbiome of the rectum differs from the microbiome of the appendix. Only one pair of rectal-appendix samples from the same subject (subject 10; red arrows) clustered together. The appendix cluster (green box) was composed almost entirely (12 out of 13 samples) of appendicitis samples, both non-perforated and perforated, suggesting that the appendix microbiome associated with appendicitis differs from the microbiome of the normal appendix. For the rectal sample cluster, 8 samples out of 10 samples from patients with appendicitis, both non-perforating and perforating, clustered together, suggesting that the microbiome of the rectum in patients with appendicitis is distinct from the microbiome of the rectum from patients without appendicitis. Samples are listed by ID number, SnX/Y, where n is the subject identification number, X describes the body site (A for appendix, R for rectum), and Y describes the patient's diagnosis (N for normal appendix, NP for an appendix with non-perforating appendicitis, and P for an appendix with perforating appendicitis).

The similarity in microbial composition among the samples analyzed in the present study was further compared using the Bray-Curtis algorithm [32], [33], an abundance-weighted measure of how similar two communities are in terms of their taxonomic composition. Communities were clustered using an average-linkage algorithm, and the results are presented in the cluster dendogram in Figure 2. Out of the 37 samples analyzed (16 rectal samples and 20 appendix samples), 13 appendix samples clustered together (green box in Figure 2). The appendix cluster was composed almost entirely (12 samples out of 13 samples belonging to that cluster) of samples from patients with appendicitis, both non-perforated and perforated appendicitis, suggesting that the appendix microbiota associated with appendicitis differs from the normal appendix. A second group of 10 samples, composed entirely of rectal samples (blue oval in Figure 2), clustered separately. For the rectal sample cluster, 8 samples out of 10 samples belonging to that cluster were from appendicitis samples, from patients with both non-perforated and perforated appendicitis, suggesting that an alteration in the appendix microbiota of patients with appendicitis is reflected in an corresponding alteration in the microbiota of the rectum. There was only one pair of rectal-appendix samples from the same subject, who did not have appendicitis (subject 10; red arrows in Figure 2) that clustered together. None of the other paired appendix and rectal samples from other patients clustered together, reinforcing the observation that the appendix and rectal microbiota have significant differences.

Taxonomic assignments of the 16S sequences revealed 290 different bacterial taxa across all the samples (Tables 1–5). The relative Phyla and Genus abundance in each sample are shown in Figures S2 and S3 in File S1. The Metastats program was used for detection of differentially abundant taxa between the appendix and rectal sites, between the appendix of patients with and without appendicitis, and between the rectum of patients with and without appendicitis (Table 2). In patients without appendicitis we observed three taxa with a statistically significant increased presence in the normal appendix compared to corresponding rectal samples: *Fusibacter* (p = 0.009), *Selenomonas* (p = 0.026), and *Peptostreptococcus* (0.049). In patients without appendicitis we also observed a statistically significant increase in abundance in seven taxa in the rectum compared to the appendix: *Frankineae* (p = 0.019), *Dyadobacter* (p = 0.019), *Actinomycineae* (p = 0.033), *Curvibacter* (p = 0.042), *Melissococcus* (p = 0.042), *Variovorax* (p = 0.042) and *Larkinella* (p = 0.042).

**Table 2:** Bacterial Genera with Significantly Different Abundance in the Normal Appendix vs. Normal Rectum.

Bacteria	Rectum	Appendix	P-value
***Fusibacter***	0	0.12%+/−0.07%	**0.009**
***Frankineae***	0.008%+/−0.008%	0	**0.019**
***Dyadobacter***	0.008%+/−0.008%	0	**0.019**
***Selenomas***	0	0.03%+/−0.02%	**0.026**
***Actinomycineae***	1.66%+/−0.92%	0.065+/−0.025%	**0.033**
***Melissococcus***	0.007%+/−0.007%	0	**0.042**
***Curvibacter***	0.007%+/−0.007%	0	**0.042**
***Variovorax***	0.007%+/−0.007%	0	**0.042**
***Larkinella***	0.007%+/−0.007%	0	**0.042**
***Peptostreptococcus***	0.016%+/−0.016%	0.32%+/−0.19%	**0.049**

*Relative abundance percentage (+/− standard error) of taxa in the normal rectum and normal appendix*. The normal appendix had 3 bacteria with significantly elevated abundance compared to the normal rectum.

**Table 3:** Bacterial Genera with Significantly Different Abundance in the Normal Appendix vs. the Appendix in Appendicitis Patients.

Bacteria	Normal Appendix	Appendicitis	P-value
***Peptostreptococcus***	0.32%+/−0.19%	5.07%+/−1.2%	**0.0003**
***Bilophila***	0.07%+/−0.06%	0.88%+/−0.21%	**0.0003**
***Parvimonas***	4.38%+/−3.61%	23.9%+/−5.33%	**0.003**
***Paenibacillaceae 1***	0.03%+/−0.03%	0	**0.005**
***Porphyromonas***	0.58%+/−0.43%	4.56%+/−1.54	**0.010**
***Bulleidia***	0.70%+/−0.45%	3.79%+/−1.21%	**0.012**
***Mogibacterium***	0.003%+/−0.002%	0.04%+/−0.01%	**0.012**
***Fusobacterium***	1.04%+/−0.67%	3.21%+/−0.69	**0.018**
***Acidobacteriaceae Gp4***	0.019%+/−0.019%	0	**0.018**
***Pseudocardineae***	0.026%+/−0.026%	0.00105%+/−0.00105%	**0.019**
***Bergeyella***	0.0086%+/−0.0086%	0.0019%+/−0.0019%	**0.019**
***Aminobacterium***	0.004%+/−0.004%	0.13%+/−0.05%	**0.019**
***Proteus***	0	0.015%+/−0.015%	**0.028**
***Actinomycineae***	0.060%+/−0.025%	1.42%+/−0.65%	**0.028**
***Anaerovorax***	0.14%+/−0.08%	0.37%+/−0.08%	**0.042**
***Rhizobium***	0.026%+/−0.026%	0.0017%+/−0.0017%	**0.045**
***Anaerofilum***	0.01%+/−0.0044%	0.04%+/−0.013%	**0.045**

Taxa with significant relative abundance percentage differences in the normal vs. diseased appendix. Relative abundance (percent, +/− standard error) of bacteria in the normal appendix and appendicitis. The normal appendix had 5 bacteria with significantly elevated abundance compared to the diseased appendix.

**Table 4:** Bacterial Genera with Significantly Different Abundance in the Rectum of Patients With and Without Appendicitis.

Bacteria	Rectum w/o Appendicitis	Rectum w/Appendicitis	P-value
***Bulleidia***	0.0021%+/−0.0021%	0.0238%+/−0.11%	**0.034**
***Porphyromonas***	0.103%+/−0.091%	0.65%+/−0.23%	**0.026**
***Dialister***	0.066%+/−0.037%	0.77%+/−0.23%	**0.003**

Relative abundance (percent, +/− standard error) of taxa in the rectal samples from patients without appendicitis and rectal samples with appendicitis.

**Table 5:** Bacterial Genera with Significantly Different Abundance in the Appendix of Patients with Perforated Appendicitis vs. Appendicitis Without Perforation.

Bacteria	Perforated Appendicitis	Appendicitis	P-value
***Bulleidia***	5.72%+/−1.72%	0.88%+/−0.58%	**0.005**
***Fusibacter***	0.29%+/−008%	0.05%+/−0.04%	**0.005**
***Prevotella***	7.66%+/−1.82%	2.77%+/−1.53%	**0.022**
***Porphyromonas***	6.50%+/−2.32%	1.65%+/−0.99%	**0.032**
***Dialister***	0.97%+/−0.30%	0.26%+/−0.23%	**0.003**

Bacteria with significant relative abundance percentage differences in appendix samples from patients with perforated vs. non perforated appendicitis.

In comparing patients with and without appendicitis, we found that five taxa showed a statistically significant increase in the normal appendix when compared to diseased appendices: *Paenibacillaceae* (p = 0.005), *Acidobacteriaceae* GP4 (p = 0.019), *Pseudonocardinae* (p = 0.019), *Bergeyella* (p = 0.019) and *Rhizobium* (p = 0.045) (Table 3). When comparing normal appendices to diseased samples, we found twelve taxa with increased abundance in appendicitis: *Peptostreptococcus* (p = 0.0003), *Bilophila* (p = 0.0004), *Bulleidia* (p = 0.012), *Fusobacterium* (p = 0.018), *Parvimonas* (p = 0.003), *Mogibacterium* (p = 0.012), *Aminobacterium* (p = 0.019), *Proteus* (p = 0.028), *Actinomycineae* (p = 0.028), *Anaerovorax* (p = 0.041), *Anaerofilum* (p = 0.045), *Porphyromonas* (p = 0.010) (Table 3).

Comparing the taxa found in the rectal samples between the patients with and without appendicitis, we found three taxa with increased abundance in the rectal samples of those with appendicitis: *Bulleidia* (p = 0.034), *Dialister* (p = 0.003) and *Porphyromonas* (p = 0.026) (Table 4). These taxa overlap with those taxa identified in the appendix, and the finding suggests that there may be a microbial signal evident in rectal samples indicative of appendicitis.

Looking more closely at the appendicitis cases, we compared non-perforated and perforated samples to determine whether any flora were over or under-represented in the most severe form of appendicitis, perforated appendicitis. We found that five genera showed a significant increase in the perforated group: *Bulleidia* (p = 0.004), *Fusibacter* (p = 0.005), *Prevotella* (p = 0.021), *Porphyromonas* (p = 0.030), *Dialister* (p = 0.035) (Table 5). The observation that taxa found to have increased abundance in the most severe form of appendicitis includes taxa that were also found to be increased in abundance in the rectums of appendicitis patients compared to the rectums of patients without appendicitis further strengthens the model that microbial analysis of rectal samples can provide helpful diagnostic insights into disease in the appendix.

**Figure 3:**
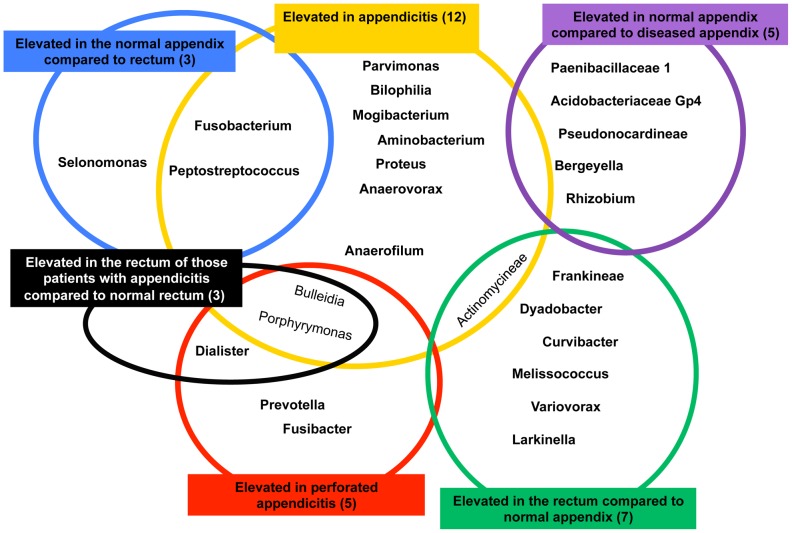
Schematic of Bacterial Genera with Significant Differences in Abundance in Appendix and Rectum, in Patients with and without Appendicitis. Top row, left to right: bacteria with elevated abundance in the normal appendix compared to the rectum, elevated abundance in appendicitis and elevated abundance in the normal appendix compared to the diseased appendix (appendicitis); Bottom row, left to right: Elevated abundance in the rectum of patient's with appendicitis compared to those with normal rectum samples, elevated abundance in perforated appendicitis compared to non perforated appendicitis and elevated abundance in the normal rectum compared to the normal appendix. The figure lists the taxa in each category; the numbers in parentheses for each heading lists the number of genera in that category.

Figure 3 shows a schematic of the genera showing significant differences comparing the appendix and rectal sites and comparing patients with and without appendicitis. We found no significant differences in relative abundance across gender, race or age.

## Discussion

Appendicitis is the most common surgical emergency. It will affect approximately 8% of persons living in Western countries at some point in their lifetimes. Despite the frequent occurrence of appendicitis, our understanding of the normal function and physiology of the appendix remains limited. While the findings we present are more extensive than previously published studies that used more limited culture techniques or FISH, our results are consistent with those previous results, which identified *Bilophilia* and *Fusobacterium* in the appendix via culture techniques and FISH respectively [21], [23]. Our 16S rRNA gene sequencing-based study results are also are consistent with the identification of *Fusobacterium* and *Parvimonas* as notable genera in the appendix microbiota [22], but the results we present also identified additional bacterial genera, not previously reported, which are found in the microbial community of the appendix, healthy and diseased. The first reported gene sequencing study examined those patients being evaluated for appendicitis while the current study observed those being evaluated for appendicitis as well as those without appendicitis, enabling a comparison to the normal appendix microbiota. It might have been desirable from a purely scientific point of view to study patients who had not been treated with antibiotics, since antibiotics could conceivably distort the composition of the appendiceal microbiota; however this would have been ethically impossible because antibiotics are considered an important element of the standard-of-care treatment of patients with suspected appendicitis, and for any patient undergoing laparotomy or laparoscopy with a surgical resection of the GI tract. While antibiotics could have altered the composition of the microbiota it is important to note that all patients were given antibiotics: patients with suspected appendicitis who were found to have appendicitis by pathologic examination, patients with clinically suspected appendicitis who were found to not have appendicitis by pathologic examination, and patients who underwent incidental appendectomy, since it is standard-of-care to give patients perioperative prophylactic antibiotics. In addition, since our study employed DNA sequence analysis of the relatively short 16S rRNA gene, not living bacterial cells, since DNA is reasonably stable, and since the time between antibiotic administration and surgery was short, it is unlikely that the administration of antibiotics substantially altered our findings. Another possible confounder is the single 5 month old infant who was included in the control group, and who underwent surgery to rule out malrotation. Since the infant GI microbiota changes significantly during development [34], the microbiota of a 5 month old may not be strictly comparable to the microbiota of an older child or an adult, but we do not believe that the inclusion of this single control subject substantially alters the conclusions of the study.

Our results show that the microbial community of the appendix is distinct from the microbial community observed in the rectal samples, suggesting that the appendix and rectum are two quite different environments, and hence support two different microbial communities. Comparing the normal appendix to the normal rectum, the normal appendix exhibits an elevated abundance of *Selenomonas*, *Fusobacterium*, and *Streptococcus*, while the normal rectum has an increased abundance of *Frankineae*, *Dyadobacter*, *Curvibacter*, *Melissococcus*, *Variovorax*, *Larkinella*, and *Actinomycineae* compared to the normal appendix. Interestingly, *Selenomonas*, in the family Veillonellaceae is perhaps best known as a colonizer of the anaerobic environment of the ruminant rumen and guinea pig cecum and while not fibrinolytic itself works in synergy with other fibrinolytic bacteria to promote dietary fiber digestion [35]. *Selenomonas* is also known as a genus that colonizes gingival pockets and is associated with severe periodontitis [36]. If the model that the appendix serves as a reservoir for beneficial microbiota is correct, then given the observation that GI microbial metabolism serves as a source of small, but significant fraction of human nutrition, then *Selenomonas* provided by the appendix may be partially responsible. *Fusobacterium* is a well-known colonizer of mammalian mucous membrane compartments. Some *Fusobacterium sp* can ferment carbohydrates and amino acids to produce butyrate and acetic acids [37]. *Fusobacterium sp* can also, however, cause invasive infections, have been implicated in periodontal disease and are an important agent in Lemierre's syndrome [38], [39]. *Peptostreptococcus* is an anaerobic, typically commensal species in humans that lives predominantly in the mouth, skin, gastrointestinal, vagina and urinary tracts. This species can become pathogenic under immunosuppressed or traumatic conditions [40], [41].

While the microbial species identified as having different abundances in the normal and diseased appendix are interesting and could conceivably be responsible either for the disease itself or sequellae associated with the disease, it is important to note that our study identifies associations only, and cannot definitively assign causality. Another important consideration is that some of the taxa we identified as being differentially present in the different specimen types and/or disease states were of relatively low abundance, which may make it more challenging to determine the exact contribution these low abundant taxa make to the pathogenesis of appendicitis or to the diagnosis of appendicitis. However, it is important to note that presence at low abundance certainly does not rule out the possibility that a microbe plays an important part in disease pathogenesis. For example, in an experimental model of *Shigella* infection, *Shigella* was by far a minority taxon in the GI tract, even though clinical effects were clearly evident [42].

Much work has studied the microbial communities of feces or the lower GI tract for clues to the pathogenesis of disease in more proximal regions of the GI tract [29], [43], [44]. This is mainly because fecal material can be collected easily, noninvasively and contains a large number of microbial cells. While these studies have yielded important information, our findings that the community observed in the rectum differs substantially from the community observed in the appendix suggests that the rectal microbial community may not be a completely valid window into the microbial physiology of more proximal areas of the GI tract. This may need to be taken into account in designing future, more extensive studies of the lower GI tract microbiota.

The bacteria in the normal and diseased appendix differ from those isolated in normal rectal samples with the exception of *Actinomycineae*, which was found to be significantly abundant in both appendicitis and normal rectal samples. Interestingly, two of the bacteria identified in non-perforated appendicitis were also identified in significant quantities in corresponding rectal samples (*Bulleidia* and *Porphyromonas*) and three of the five bacteria identified in perforated appendicitis were identified in corresponding rectal samples (*Bulleidia*, *Porphyromonas and Dialister*). Thus, identification of *Bulleidia*, *Porphyromonas* and *Dialister* in the rectum may be a secondary indicator of appendicitis, an indicator that could conceivably be used to develop diagnostics for appendicitis, although the clinical significance of such indicator bacterial species would have to be confirmed by larger scale prospective clinical studies.

The bacteria identified in the normal and diseased appendix include organisms several of which are found in the healthy human microbiota in many sites, including the gastrointestinal tract, upper respiratory tract, vagina and oral cavity. If the human host is compromised (in this case, secondary to appendiceal obstruction causing bacterial overgrowth), many of these organisms may be involved in bacteremia and septicemia (ie. *Peptostreptococcus, Bilophila, Fusobacterium, Parvimonas, Proteus, Dialister, Prevotella, Porphyromas*) [45], [46]. A study by Dharmani et al showed that *Fusobacterium nucleatum* (a normal inhabitant of the human mouth and gut) derived from the inflamed intestines of Crohn's disease patients evoked significantly greater gene expression of mucin and tumor necrosis factor alpha gene than other bacteria isolated from the non-inflamed gut in human colonic epithelial cells [47]. Another study by Qin et al showed that 155 bacterial species of the fecal microbiota were found to be present at significantly different relative abundance between patients with Crohn's disease, ulcerative colitis and healthy control patients [48], [49]. While no specific mechanisms have been identified that implicate the bacteria identified in our current study as contributing to the disease characteristics of appendicitis, the studies point to instances in which specific bacteria evoke unique inflammatory responses characteristic of the symptoms in inflammatory bowel disease. It must be noted, however, that our observations only report associations and so cannot definitively establish a causal relationship. More studies will be needed to elucidate the specific bacterial mechanisms that contribute to appendicitis pathogenesis.

It is possible that obstruction from a fecalith or prominent lymphoid tissue leads to alterations in the microbiota of the normal appendix, leading to colonization by organisms associated with appendicitis, which then produce the clinical sequelae of inflammation, compromised perfusion and, ultimately, perforation. Larger scale studies that examine abundance and virulence factor shifts in the normal and diseased appendix are needed to further elucidate the complete process of appendicitis pathogenesis.

Our finding that some of the organisms that have increased abundance in the diseased appendix are also present in increased abundance in the rectal samples of those with appendicitis raises some intriguing questions. The data suggest that with appendicitis, organisms linked to appendicitis are not confined to the appendix, perhaps because the normal physiology of the appendix is compromised. The data further suggest that the detection of appendicitis-associated microbes in the rectum may represent a remote microbial signal of appendicitis, a signal that could perhaps be exploited to develop rectal microbe-based diagnostics for appendicitis.

Additional limitations of our study include its sample size and the administration of antibiotics. More samples, particularly more normal samples, are needed in future studies to better characterize the normal microbiome of the appendix. While each participant received antibiotics regardless of surgical indication, (ie. appendicitis vs. appendectomy incidental to surgery for other indications), the impact of these antibiotics on the composition of the appendiceal and rectal microbiota is unclear. It is not ethically possible to withhold antibiotics from a patient with suspected appendicitis about to undergo abdominal surgery, so it will be impossible to definitively assess the microbiota of the appendix in patients who have not received antibiotics.

Looking forward, study of specific pathogens identified through culture-independent 16S rRNA sequence determination and analysis could not only help to further characterize the normal microbiota of the appendix and other gastrointestinal organs, but may lead to enhanced preoperative evaluation, differential diagnosis, and expedited care in the treatment of appendicitis and other medical/surgical conditions.

## Supporting Information

File S1 **This file contains Figure S1–Figure S3.** Figure S1, Bacterial Community Richness and Diversity Estimators by Sample. The figure shows, for each sample, the sample type (appendix or rectum), the diagnosis (normal, appendicitis, perforated appendicitis), the observed number of OTUs, and the Chao1, ACE (richness), Shannon (diversity), and Simpson values. Figure S2, Bacterial community composition at the phylum level. The figure shows the bacterial relative abundance (fraction of total sequence reads assigned to each of the bacteria phyla listed in the legend on the right side) for each of the samples studied. The sample names follow the convention used in Figure 1: Samples are listed by ID number, SnX/Y, where n is the subject identification number, X describes the body site (A for appendix, R for rectum), and Y describes the patient's diagnosis (N for normal appendix, NP for an appendix with non-perforating appendicitis, and P for an appendix with perforating appendicitis). Figure S3, Bacterial community composition at the genus level. The figure shows the bacterial relative abundance (fraction of total sequence reads assigned to each of the bacteria genera listed in the legend on the right side). The sample names follow the convention used in Figure 1: Samples are listed by ID number, SnX/Y, where n is the subject identification number, X describes the body site (A for appendix, R for rectum), and Y describes the patient's diagnosis (N for normal appendix, NP for an appendix with non-perforating appendicitis, and P for an appendix with perforating appendicitis).
(DOC)
