# Supplementary material for: Culture-Independent Evaluation of the Appendix and Rectum Microbiomes in Children with and without Appendicitis
Source: PLoS One. 2014 Apr 23;9(4):e95414. doi: 10.1371/journal.pone.0095414 (PMC3997405; doi:10.1371/journal.pone.0095414)

***SUPPORTING DATA***

***Supporting Figure S1.*** Bacterial Community Richness and Diversity Estimators by Sample.

The figure shows, for each sample, the sample type (appendix or rectum), the diagnosis (normal, appendicitis, perforated appendicitis), the observed number of OTUs, and the Chao1, ACE (richness), Shannon (diversity), and Simpson values.

***Supporting Figure S2.*** Bacterial community composition at the phylum level. The figure shows the bacterial relative abundance (fraction of total sequence reads assigned to each of the bacteria phyla listed in the legend on the right side) for each of the samples studied. The sample names follow the convention used in Figure 1: Samples are listed by ID number, SnX/Y, where n is the subject identification number, X describes the body site (A for appendix, R for rectum), and Y describes the patient’s diagnosis (N for normal appendix, NP for an appendix with non-perforating appendicitis, and P for an appendix with perforating appendicitis).

***Supporting Figure S3.*** Bacterial community composition at the genus level. The figure shows the bacterial relative abundance (fraction of total sequence reads assigned to each of the bacteria genera listed in the legend on the right side). The sample names follow the convention used in Figure 1: Samples are listed by ID number, SnX/Y, where n is the subject identification number, X describes the body site (A for appendix, R for rectum), and Y describes the patient’s diagnosis (N for normal appendix, NP for an appendix with non-perforating appendicitis, and P for an appendix with perforating appendicitis).

**Supporting Figure S1.** Bacterial Community Richness and Diversity Estimators by Sample.


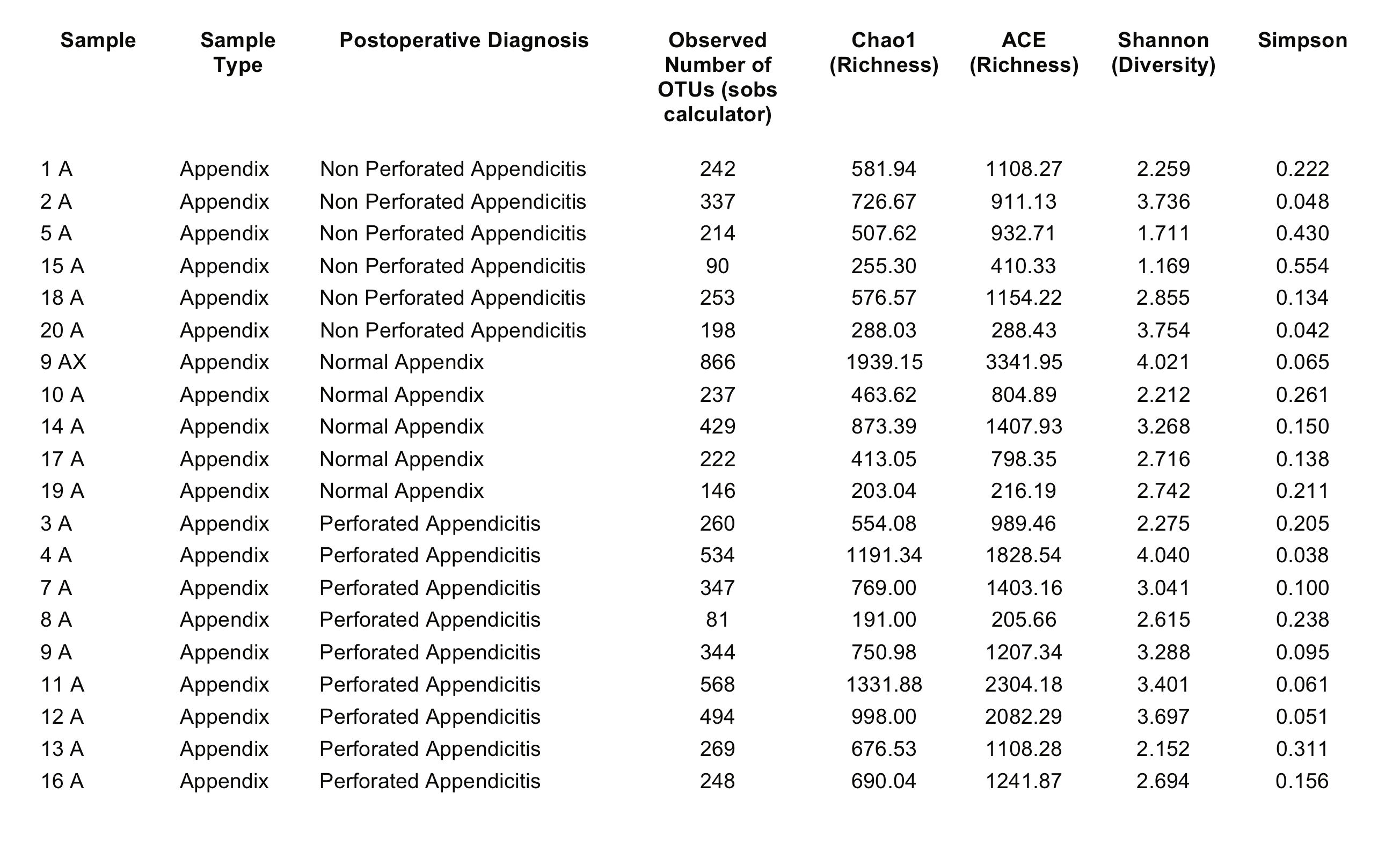


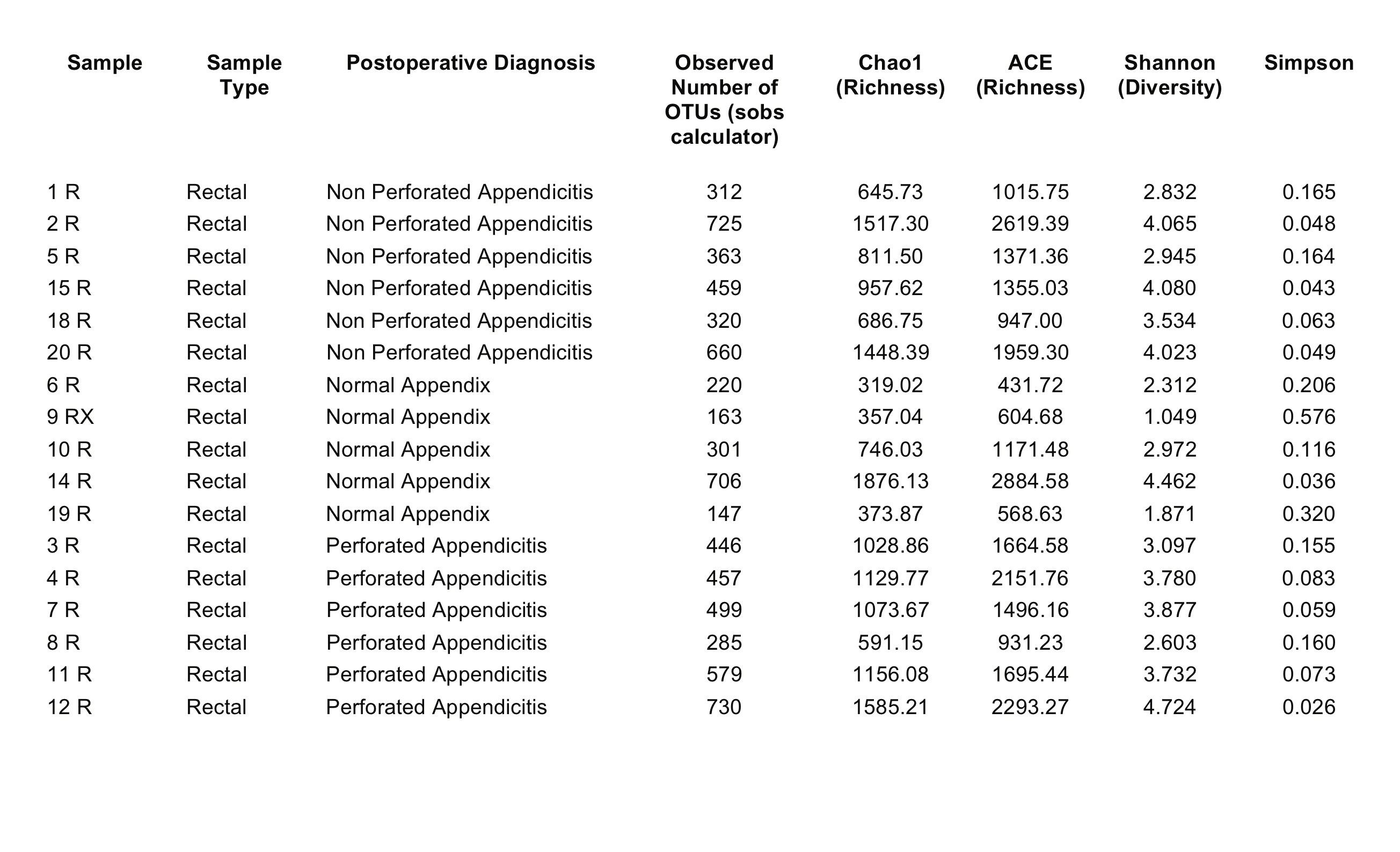


***Supporting Figure S2.*** Sample by sample microbial Phyla abundance.

**
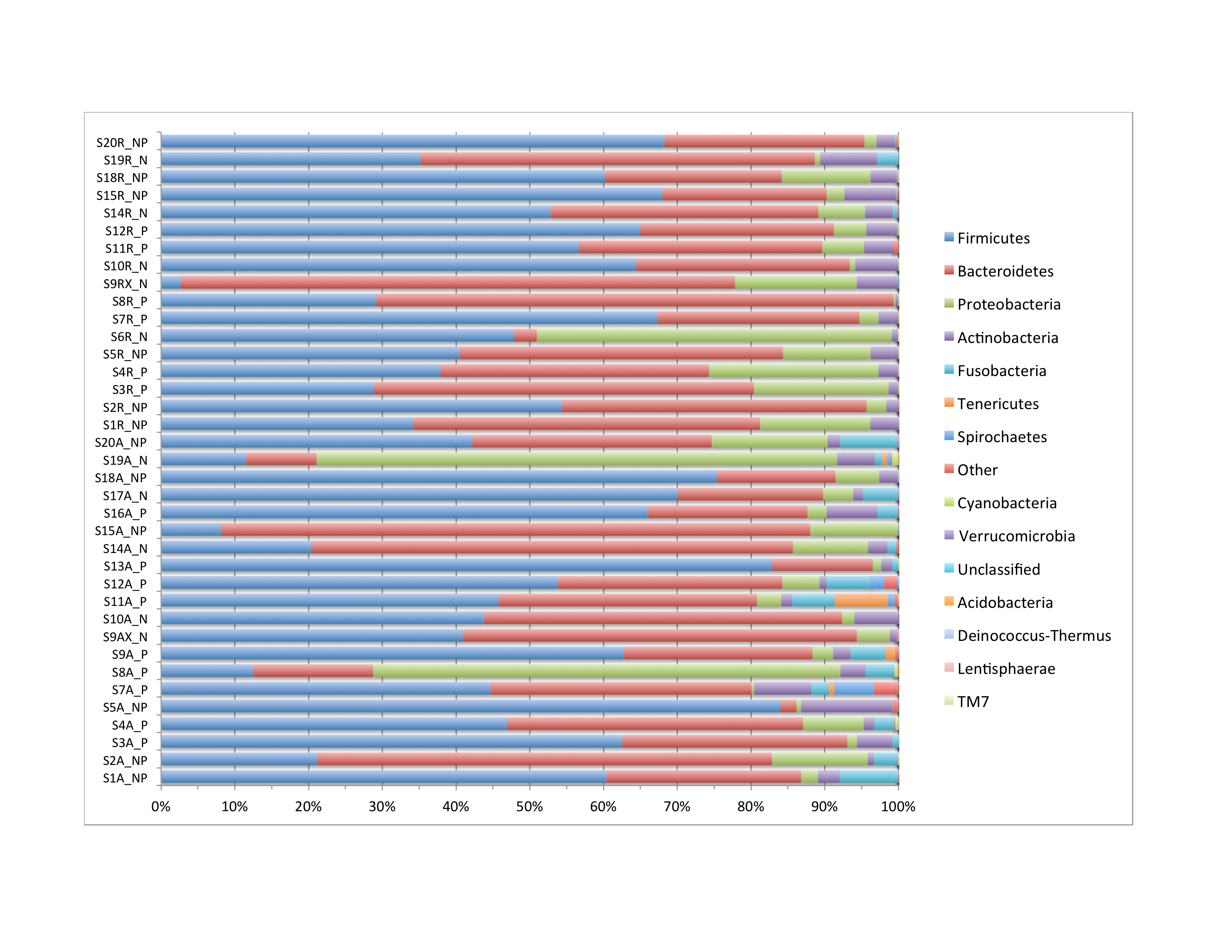
**

***Supporting Figure S3.*** Sample by sample microbial Genus abundance.


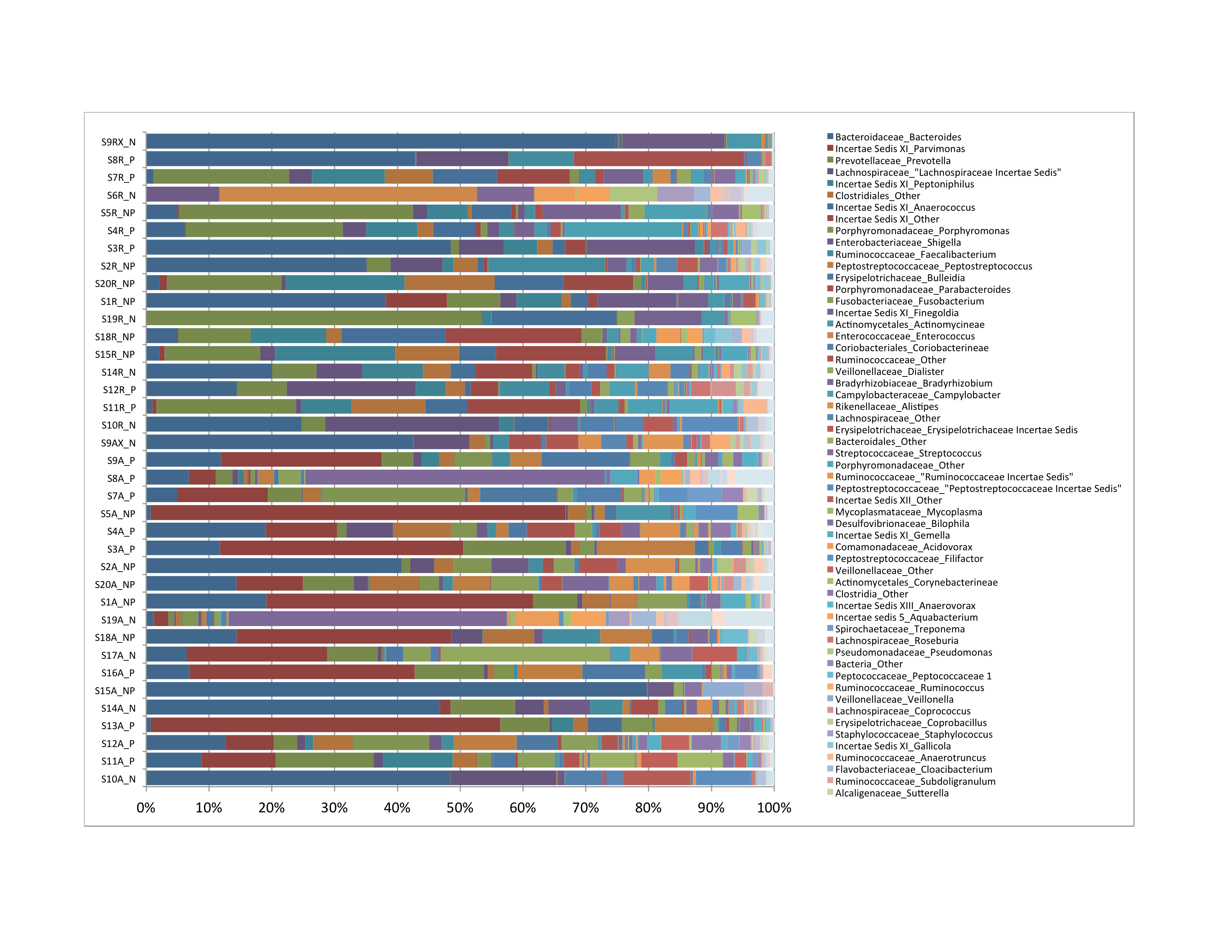

Supplement: File S1 — This file contains Figure S1–Figure S3. Figure S1, Bacterial Community Richness and Diversity Estimators by Sample. The figure shows, for each sample, the sample type (appendix or rectum), the diagnosis (normal, appendicitis, perforated appendicitis), the observed number of OTUs, and the Chao1, ACE (richness), Shannon (diversity), and Simpson values. Figure S2, Bacterial community composition at the phylum level. The figure shows the bacterial relative abundance (fraction of total sequence reads assigned to each of the bacteria phyla listed in the legend on the right side) for each of the samples studied. The sample names follow the convention used in Figure 1: Samples are listed by ID number, SnX/Y, where n is the subject identification number, X describes the body site (A for appendix, R for rectum), and Y describes the patient's diagnosis (N for normal appendix, NP for an appendix with non-perforating appendicitis, and P for an appendix with perforating appendicitis). Figure S3, Bacterial community composition at the genus level. The figure shows the bacterial relative abundance (fraction of total sequence reads assigned to each of the bacteria genera listed in the legend on the right side). The sample names follow the convention used in Figure 1: Samples are listed by ID number, SnX/Y, where n is the subject identification number, X describes the body site (A for appendix, R for rectum), and Y describes the patient's diagnosis (N for normal appendix, NP for an appendix with non-perforating appendicitis, and P for an appendix with perforating appendicitis). (DOC) [file pone.0095414.s001.doc]
